# An observational 3-year follow-up study for postoperative renal function changes assessed by ^99 m^Tc-DTPA scintigraphy and predictive factors after miniaturized percutaneous nephrolithotomy and retrograde intrarenal surgery

**DOI:** 10.1186/s12894-025-01761-3

**Published:** 2025-04-29

**Authors:** Hyomyoung Lee, Yerzhan Sharapatov, Hyeji Park, Christine Joy Castillo, Majed Alharthi, Mohammad Zogan, Sung Yong Cho

**Affiliations:** 1 Department of Urology, Seoul National University Hospital, Seoul National University College of Medicine, 101, Daehak-ro, Jongno-gu, Seoul, 03080 Republic of Korea; 2 Department of Urology, Seoul National University College of Medicine, Seoul, Republic of Korea; 3 Department of Urology, Astana Medical University, Astana City, Kazakhstan; 4 Department of Urology, Jose R. Reyes Memorial Medical Center, Sta. Cruz, Manila, Philippines; 5 Department of Urology, King Fahd General Hospital, Jeddah, Saudi Arabia; 6 Department of Urology, Prince Mohammed Bin Nasser Hospital, Jazan, Saudi Arabia

**Keywords:** Urinary calculi, Minimally invasive surgical procedures, Kidney function tests, Kidney calculi

## Abstract

**Background:**

This study aims to investigate changes in relative renal function three years after miniaturized percutaneous nephrolithotomy (mini-PCNL) and retrograde intrarenal surgery (RIRS) and to identify significant predictors associated with renal function aggravation.

**Methods:**

From 2019 to 2023, 355 patients aged 20 and above who underwent mini-PCNL or RIRS for the treatment of renal stones > 10 mm in maximal diameter were prospectively included in this study, with those who had separate renal function serially traced being included. Among them, 93 patients were included in the analysis. Renal function was evaluated using 99 mTc-DTPA before surgery, and at 3 months, 1, 2, and 3 years postoperatively.

**Results:**

A difference in preoperative renal function of > 10% between the contralateral and operative sides was observed in 79 patients (84.9%). Among those in the abnormal renal function group, 42 patients (53.2%) showed stability, 31 (39.2%) showed aggravation, and 6 (7.6%) showed improvement in renal function at postoperative 3 years. Functional changes did not differ significantly between the types of surgery. Significant predictors of renal function aggravation included higher preoperative creatinine levels, preoperative hydronephrosis, and S-ReSC > 3.

**Conclusion:**

A key point of this study is that it is important to explain to patients who do not show recovery within 1 to 2 years that there is a 42.1% chance their renal function may deteriorate over time. Clinicians should be particularly attentive to renal function in patients with higher preoperative creatinine levels, preoperative hydronephrosis, and S-ReSC > 3, requiring closer monitoring and management.

## Background

The prevalence of renal stones ranges from 1 to 13% in the general population, and it is a common urological disease with an increasing incidence worldwide [1, 2]. Intervention for renal stones is recommended in cases of urinary tract obstruction, hematuria, or unmanageable pain, presence of growing stones, stones persisting for more than 2 years, or in patients at increased risk of stone formation or urinary tract infections [3–5]. However, this recommendation in the current guidelines has limitations as it does not consider renal functional assessment yet. Given that urolithiasis is a known risk factor for renal function deterioration, data on renal function is important to evaluate long-term outcomes of stone management interventions [4, 5].

Among various treatment options for renal stones, mini-PCNL and RIRS are known to be more effective and have fewer complications compared to conventional surgical methods [6, 7]. In recent years, there has been increasing interest in understanding the changes in renal function following minimally invasive renal surgery. Bilen et al. observed stable or improved estimated glomerular filtration rate (eGFR) at three months after conventional PCNL in patients with late-stage chronic kidney disease (CKD) [8]. Studies have also demonstrated improvement in relative renal function at three months post-mini-PCNL and RIRS using diethylenetriamine penta-acetic acid (^99 m^Tc-DTPA) and technetium- 99 m dimercaptosuccinic acid (^99 m^Tc-DMSA) [9]. Jung et al. assessed relative renal function using DTPA one year after RIRS and mini-PCNL and found no significant difference in functional changes based on surgical type [10]. However, to our knowledge, there are currently no data regarding the mid-to-long -term impact of minimally invasive renal surgery on relative renal function.

Our hypothesis was that mini-PCNL and RIRS would have similar effects on long-term renal function. Additionally, our aim was to examine how predictors of renal function change when follow-up is extended from 1 to 3 years. This study investigated changes in relative renal function three years after mini-PCNL and RIRS, assessed using ^99 m^Tc-DTPA, and identify significant predictors associated with renal function deterioration. By investigating predictive factors influencing renal function aggravation, we can understand the long-term effects of these procedures on renal function. Therefore, optimizing patient selection, treatment strategies, and postoperative management protocols can improve clinical outcomes in stone management.

## Methods

### Patients

From 2019 to 2023, out of 1,973 cases, 355 patients aged 20 and above who underwent mini-PCNL or RIRS for the treatment of renal stones > 10 mm of the maximal diameter were prospectively included in this study, with only those who had separate renal function serially traced being included. This study was conducted as a retrospective analysis of prospectively collected data.

This study was approved by the Institutional Review Board (IRB) of Seoul National University Hospital (IRB No.1901–104–1005). The decision to undergo surgery was based on the EAU guidelines [3]. Exclusion criteria for the analysis included febrile urinary tract infection, age under 20, bleeding tendency, pregnancy, urogenital anomalies, solitary kidney, bilateral stones, concomitant use of flexible ureteroscopy for mini-PCNL, preoperative hydronephrosis with complete urinary tract obstruction, and three or more percutaneous tracts. Informed consent was obtained from all patients for their participation in the study, the use of their medical data for research purposes, and for undergoing laboratory tests and DTPA renograms.

### Study design

Preoperative medical history taking and physical examinations were conducted, along with assessments of Hemoglobin levels (mg/dL), Creatinine levels, plain radiography (kidneys, ureters, bladder [KUB]), Non-contrast computed tomography (CT), and ^99 m^Tc-DTPA scintigraphy. eGFR was calculated using the Modification of Diet in Renal Disease (MDRD) Eq. [11]. When eGFR is < 60 mL/min/1.73 m^2^, the Chronic Kidney Disease Epidemiology Collaboration (CKD-EPI) equation was used instead of MDRD Eq. [12].

Stone characteristics were determined as follows: Stone volume was calculated as the sum of the volumes of each stone, with stone volume calculated using the formula 0.523 × length × width × height (mm^3^). Seoul National University Renal Stone Complexity (S-ReSC) scores were assigned based on the categorization of the kidney collecting system, including renal pelvis, major calyces, and minor calyces [13].

Individual renal function was evaluated in terms of the surgical side (Fn_Op) and contralateral side (Fn_Con). A normal group was defined as having a difference of ≤ 10% in the measured absolute values between the contralateral and surgical sides (GAP, Fn_Con/Fn_Op). An abnormal group was defined as having a GAP of more than 10%. Since there is no established cut-off to differentiate normal from abnormal in renal function scans, and considering the margin of error in the test results, values close to 50% were arbitrarily defined as a range of 44–55%. Cases falling outside this range have been defined as outside the normal range in previous studies of the present authors [9, 14]. In the abnormal group, if the re-evaluated renal function changed by > 10% compared to the preoperative value, it was classified as"improvement"or"deterioration,"(Table 1). The 10% threshold in this study was arbitrarily defined. Deterioration of the separate renal function was defined when the difference between the postoperative Fn_Op and Fn_Con became 10% larger than that between the preoperative Fn_Op and Fn_Con. If the GAP was greater than 10% on re-evaluation but renal function did not change larger than 5%, it was defined as functionally"stable". If the renal function was greater than 5% on re-evaluation compared to the previous evaluation and the GAP becomes > 10%, it was defined as functionally"aggravated". Significant predictors of renal function changes were determined based on patient characteristics, stone data, and pre- and postoperative characteristics.

**Table 1:** Definitions for renal function assessment

Category	Definition
GAP	Difference in separate renal function between the contralateral kidney and the operative kidney
Improvement	Postoperative GAP decreases by more than 10% compared to preoperative GAP
Deterioration	Postoperative GAP increases by more than 10% compared to preoperative GAP
Stable	GAP remains over 10% but renal function changed by less than 5%
Aggravation	GAP exceeds 10% and renal function changes by more than 5% from the previous evaluation

Perioperatively parameters included operative time (minutes), stone-free rate, residual stones (defined as > 2 mm), and complications. The follow-up conducted 3 months postoperatively included KUB and CT to investigate the presence of residual stones. Stone-free status was defined as the absence of residual stones or stones < 2 mm on postoperative images at three months. The modified Clavien–Dindo system was used for classifying perioperative complications [15]. The renal function was evaluated using ^99 m^Tc-DTPA before surgery, and at 3 months, 1 year, 2 years, and 3 years postoperatively. If the ^99 m^Tc-DTPA results at 3 month, 1 or 2 years fell within the 44–55% normal range, further DTPA follow-up was discontinued, and only creatinine, GFR, and noncontrast kidney CT were monitored.

### Surgical methods

#### 1) Mini-PCNL

Patients were positioned either supine or prone and underwent mini-PCNL while under general anesthesia. The percutaneous tract establishment was performed by a single experienced urologist (CSY) using a combination of ultrasonography and fluoroscopy. Insertion into the renal pelvis through the tract was facilitated using a 0.035-mm Terumo or Zip guidewire from Terumo Group, Tokyo, Japan, in conjunction with a Superstiff guidewire from Boston Scientific, Miami, FL, USA. Tract dilation followed with an Ultraxx™ balloon dilator from Cook Medical, Bloomington, IN, USA, reaching up to 18 Fr. Subsequently, either a 15-Fr Miniature nephroscope from Richard Wolf, Knittlingen, Germany, or a 16.5-Fr MIP M nephroscopic system from Karl Storz, Tuttlingen, Germany, was inserted. Most (90%) of PCNL cases utilized a single tract ranging from 16.5 to 18 Fr. In cases where two tracts were created, the first tract ranged from 16.5 to 18 Fr, and 12 Fr, a MIP S nephroscopic system from Karl Storz was also used. Stone fragmentation was achieved using a holmium:yttrium–aluminum-garnet (YAG) laser equipped with a 550-µm fiber from either Trimedyne, Irvine, CA, USA, or Lumenis Ltd., Boston Scientific, Miami, FL, USA. If necessary, fragmented stones were extracted using a 5-Fr grasping Alligator forceps from Richard Wolf. A 6-Fr ureteral JJ stent was routinely inserted for approximately one week, followed by the placement of a 16-Fr urethral Foley catheter. In most instances, the placement of a percutaneous nephrostomy tube was unnecessary.

#### 2) RIRS

Patients positioned in the dorsal lithotomy underwent RIRS under general anesthesia. Following cystoscopic or ureteroscopic examination, an 11/13-Fr ureteral access sheath was inserted, guided by a 0.035-mm Terumo or Zip guidewire, 5-Fr ureteral catheter, and Superstiff guidewire, until reaching the ureteropelvic junction. A Flex-X2, X2S™, or Xc™ flexible ureteroscope from Karl Storz or a URF-P5™ or URF-V2™ flexible ureteroscope from Olympus, Tokyo, Japan, was then introduced through the ureteral access sheath. Stone fragmentation was performed using a holmium:YAG laser equipped with either a 365- or 200-µm laser fiber, followed by the removal of fragmented stones with a stone basket. A 6-Fr ureteral JJ stent was routinely placed post-lithotripsy, and finally, a 16-Fr urethral Foley catheter was inserted.

#### Statistical analysis

Statistical analyses were conducted using IBM SPSS Statistics version 22.0 (IBM Corp., Armonk, NY, USA). Comparative results between the two surgical groups were analyzed using either the independent t-test or Mann–Whitney U test. Categorical variables were analyzed using Chi-square and Fisher’s exact tests. Univariate and multivariate logistic regression analyses were employed, utilizing a stepwise approach to identify predictors of changes in renal function aggravation. P-value < 0.05 was considered statistically significant.

## Results

### Patient demographics and stone parameters

Table 2 provides patient demographics and stone characteristics. 262 patients had DTPA results within the normal range at the 1-year and 2-year marks, leading to the discontinuation of follow-up. Patients were included based on having complete follow-up data, including serial renal function assessments using DTPA. Of the 93 patients who were followed for three years, 61 underwent RIRS, while 32 underwent mini-PCNL. There were no significant differences between the two groups in terms of age, gender, body mass index (BMI), comorbidities, eGFR, creatinine, and hemoglobin. However, the total volume of stones and the S-ReSC score were significantly higher in the mini-PCNL group compared to the RIRS group.

**Table 2:** Patient and Stone characteristics, Surgical parameters and Renal function parameters according to the surgical method (*n* = 93)

Variable	RIRS	Mini-PCNL	*P-value*
Total patients (n,%)	61 (65.6%)	32 (34.4%)	
**Patient parameters**			
Mean age (years)	56.5 ± 11.9	55.7 ± 10.9	0.75
Gender (male)	44 (72.1%)	22 (68.8%)	0.73
Mean BMI (kg/m^2^)	25.4 ± 4.0	25.1 ± 2.8	0.67
Comorbidities (n,%)			
Diabetes mellitus	12 (19.7%)	4 (12.5%)	0.38
Hypertension	25 (41.0%)	13 (40.6%)	0.97
Preoperative Lab values			
Creatinine (mg/dL)	1.1 ± 0.3	0.9 ± 0.3	0.09
eGFR (mL/min/1.73 m^2^)	75.2 ± 20.3	83.8 ± 21.6	0.60
Hemoglobin (mg/dL)	13.5 ± 1.6	13.5 ± 1.6	0.87
**Stone parameters**			
Total number	3.1 ± 3.9	3.2 ± 2.8	0.89
Total volume (mm^3^)	1,126.31 ± 1,993.8	12,047.6 ± 29,657.1	**0.01**
S-ReSC score	2.7 ± 2.1	4.8 ± 3.4	**0.01**
Hydronephrosis (n,%)	13 (21.3%)	11 (34.4%)	0.17
**Surgical parameters**			
Operation time (min)	45.5 ± 39.9	89.0 ± 52.9	**0.01**
Stone-free rate (n,%)	58 (95.1%)	26 (81.2%)	**0.03**
Residual stones > 2 mm	3 (4.9%)	6 (18.8%)	**0.03**
Complication rate (n,%)	6 (9.8%)	5 (15.5%)	0.41
Grade I			
Fever/pain	3 (4.8%)	1 (3.1%)	
Grade II			
Acute pyelonephritis	1 (1.6%)	1 (3.1%)	
Transfusion	2 (3.2%)	1 (3.1%)	
Grade III			
Embolization	0 (0.0%)	2 (6.2%)	
Ureter stricture/fistula	0 (0.0%)	0 (0.0%)	
**Renal function parameters**			
Preoperative separate renal function (Fn_OP/Fn_Con) (%)	42.5 ± 15.3/57.5 ± 15.3	41.3 ± 19.2/58.7 ± 19.2	0.751
Change of GAP (36 mo) (%)	2.9 ± 13.9	1.1 ± 11.4	0.554
Postoperative renal function			0.469
Improved	3 (5.8%)	3 (11.1%)	
Stable	30 (57.7%)	12 (44.4%)	
Aggravated	19 (36.5%)	12 (44.4%)	

*BMI* body mass index, *GFR* glomerular filtration rate, *PCNL* percutaneous nephrolithotomy, *RIRS* retrograde intrarenal surgery, *S-ReSC* Seoul National University Renal Stone Complexity

### Surgical parameters and complications

The operation time was significantly longer in the mini-PCNL group (89.0 ± 52.9 min) compared to the RIRS group (45.5 ± 39.9 min). The stone-free rate was significantly higher in the RIRS group (95.1%) compared to the mini-PCNL group (81.2%). Residual stones were significantly more common in the mini-PCNL group (18.8%) compared to the RIRS group (4.9%). There was no significant difference in complication rates between the mini-PCNL and RIRS groups.

### Renal function parameters

**Fig. 1:**
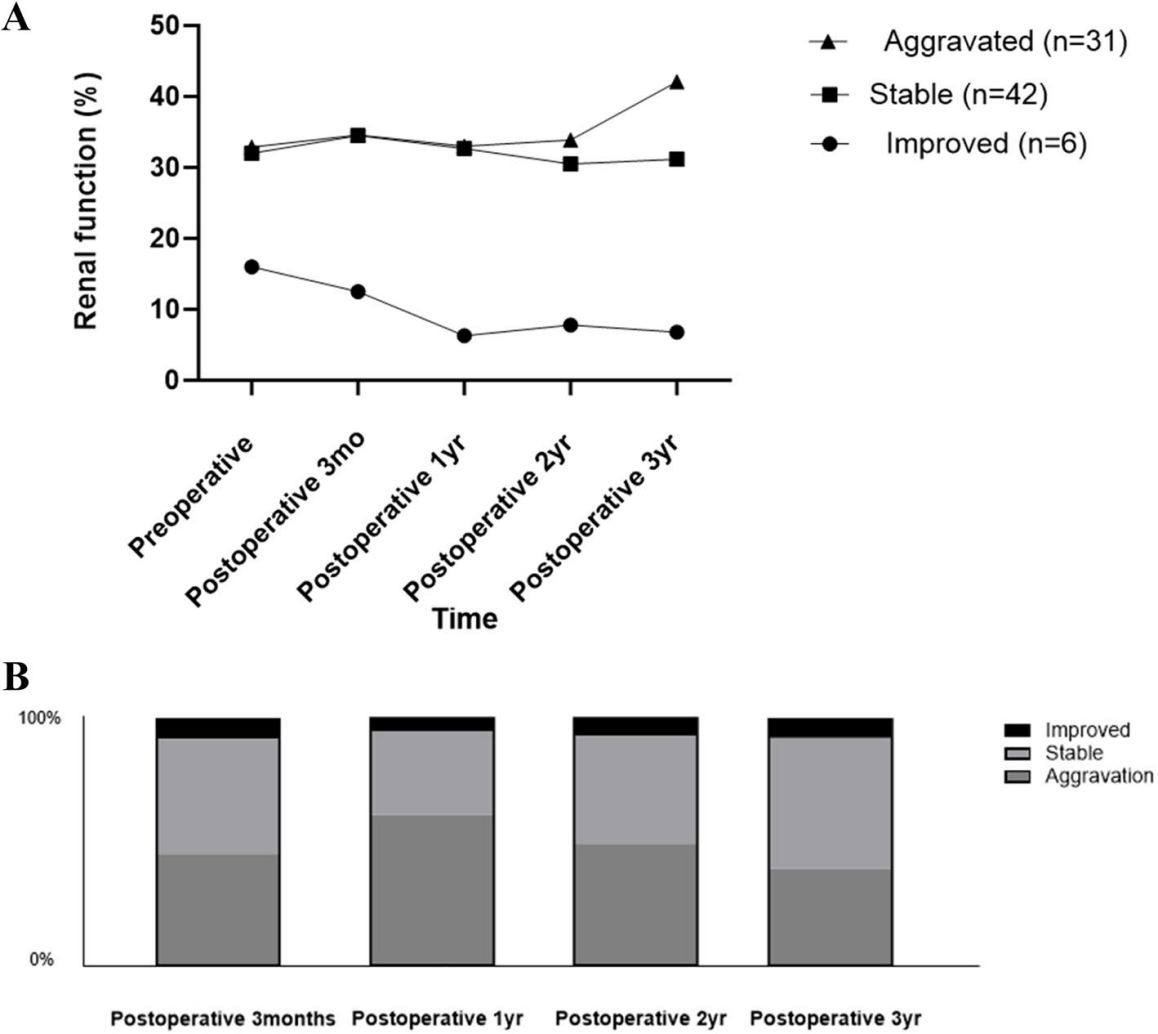
**A** GAP^#^ in the separate renal functions. ^#^GAP was defined as the separate renal function of the contralateral kidney minus that of the operative kidney. **B** Ratio of each group (Improved, Stable, Aggravation)

Out of a total of 93 individuals, 14 (15.1%) cases belonged to the normal function group. In the abnormal function group, there was no significant difference in preoperative Fn_OP between the RIRS group at 42.5 ± 15.3% and the mini-PCNL group at 41.3 ± 19.2% (Table 2). There was no significant difference in the change in GAP at 36 months postoperatively between the RIRS (2.9 ± 13.9%) group and the mini-PCNL (1.1 ± 11.4%) group. After 36 months post-surgery, the aggravation group had a 42.1% gap in separate renal function, whereas the stable group had a 31.2% gap (Fig. 1A). Among patients in the abnormal function group, 42 (53.2%), 31(39.2%), and 6 (7.6%) patients showed stability, aggravation, and improvement in renal function at postoperative 36 months, respectively (Fig. 1B).

### Prediction of aggravated renal function

The predictors of renal function aggravation at 36 months post-surgery are listed in Table 3, based on both univariate and multivariate logistic analyses. These analyses included both the group that was followed up for the entire 36 months and the group that showed normal results and was followed up only until the 1 st or 2nd year (Table 3A), as well as those followed up for the full 36 months (Table 3B).

**Table 3:** Univariate and multivariate logistic regression analysis of the significant predictors of postoperative aggravation of renal function (A) both the group that was followed up for all 36 months and the group that showed normal results and was followed up only until the 1^st^ or 2^nd^ year, (B) the group followed up for the full 36 months

(A)	*Univariate*			*Multivariate*		
	*p-value*	*OR*	*95% CI*	*p-value*	*OR*	*95% CI*
Age > 60	0.522	1.022	0.955 - 1.094			
Body mass index	0.627	0.931	0.699 - 1.241			
Diabetes mellitus	0.808	1.393	0.096 - 20.226			
Hypertension	0.869	1.151	0.215 - 6.165			
Mini-PCNL (vs RIRS)	0.127	4.578	0.648 - 32.363			
Laterality (Left)	0.310	2.287	0.463–11.295			
Previous SWL	0.391	0.463	0.080 - 2.692			
Previous stone procedure	0.216	3.154	0.511 - 19.485			
Preoperative creatinine	0.133	8.037	0.530 - 121.881	0.064†	5.451	0.906–32.791
Preoperative hydronephrosis	0.001*	34.710	4.533–265.772	0.001*	12.433	2.919–52.963
Operation time > 90 min	0.297	2.784	0.406–19.085			
Number of stones > 3	0.373	2.154	0.399–11.630			
Total stone volume > 1,000 mm^3^	0.678	0.653	0.088–4.871			
S-ReSC > 3	0.072†	5.515	0.856–35.525	0.039*	6.139	1.100–34.267
Hounsfield unit > 1,000	0.650	0.694	0.144–3.352			
** (B)**	*Univariate*			*Multivariate*		
	*p-value*	*OR*	*95% CI*	*p-value*	*OR*	*95% CI*
Age > 60	0.929	1.087	0.176 - 6.727			
Body mass index	0.232	0.837	0.625 - 1.121			
Diabetes mellitus	0.249	6.022	0.284 - 127.793	0.069†	10.902	0.831–143.096
Hypertension	0.961	1.053	0.133 - 8.306			
RIRS (vs mini-PCNL)	0.179	0.157	0.011 - 2.340	0.045^*^	0.113	0.013–0.950
Laterality (Left)	0.917	0.910	0.156 - 5.312			
Previous SWL	0.972	0.965	0.133 - 7.001			
Previous stone procedure	0.353	0.313	0.027 - 3.633			
Preoperative creatinine	0.022^*^	95.955	1.912 - 4816.390	0.003^*^	154.202	5.733–4147.921
Preoperative hydronephrosis	0.126	10.853	0.511–230.546	0.032^*^	13.428	1.256–143.509
Operation time > 90 min	0.597	2.153	0.125–37.081			
Number of stones > 3	0.488	0.452	0.048–4.263			
Total stone volume > 1,000 mm^3^	0.193	0.116	0.005–2.972	0.056†	0.104	0.010–1.060
S-ReSC > 3	0.047^*^	26.677	1.042–682.765	0.009^*^	25.234	2.203–289.027
Hounsfield unit > 1,000	0.519	1.942	0.259–14.578			
Maximal diameter of stone	0.640	1.023	0.930–1.125			
* OR *odds ratio, *CI *confidence interval, *S-ReSC *Seoul National University Renal Stone Complexity, *RIRS *retrograde intrarenal surgery, *mini-PCNL *miniaturized percutaneous nephrolithotomy, *SWL *shock wave lithotripsy						

^*^P < 0.05 as stastically significant, †*P* < 0.10 and *P* > 0.05 borderline significance.

Table 3A shows that, in the univariate analysis, the presence of preoperative hydronephrosis was the only significant predictor of renal function aggravation, while S-ReSC > 3 was borderline significant. In the multivariate analysis, the presence of preoperative hydronephrosis and S-ReSC > 3 were significant predictors, while a preoperative high creatinine level was borderline significant.

Table 3B shows that, in the univariate analysis, preoperative creatinine and S-ReSC > 3 were significant predictors of renal function aggravation. In the multivariate analysis, mini-PCNL surgery (versus RIRS), preoperative high creatinine level, the presence of preoperative hydronephrosis, and S-ReSC > 3 were significant predictors, while a high stone volume (> 1,000 mm^3^) and the presence of diabetes were borderline significant.

## Discussion

There have been studies on renal function after conventional PCNL. For example, Kukreja et al. and Canes et al. reported improved or similar renal function after PCNL in long-term follow-up [16, 17]. Similarly, Akman et al. observed improved or stable eGFR in patients treated with conventional PCNL for staghorn calculi [18]. However, these studies are limited by their reliance on serum creatinine levels as the sole indicator of renal function, lacking a more comprehensive evaluation of individual renal function, needing more assessment of separate renal function. Moreover, minimally invasive techniques, such as mini-PCNL and RIRS, have gained attention as new approaches in renal stone surgery [6, 7]. There are studies evaluating relative renal function using ^99 m^Tc-DTPA or ^99 m^Tc-DMSA after mini-PCNL and RIRS [9, 10]. However, these studies have a short follow-up period ranging from 3 to 12 months, highlighting the shortage of long-term follow-up data. Therefore, evaluating relative renal function over the long term postoperatively and gathering data on predictors of renal function deterioration would benefit clinical practice.

Given the need to monitor early renal function changes as a predictor of long-term outcomes, selecting an appropriate initial evaluation time point is crucial. Previous studies have shown that early renal function changes typically stabilize within the first few months postoperatively, making the 3-month time point a reasonable choice for the first evaluation [9, 10]. Bilen et al. also reported stable or improved eGFR at 3 months after conventional PCNL, further supporting its clinical relevance [19]. This time point enables early detection of functional changes and aligns with standard postoperative follow-up protocols.

In this study, the difference in preoperative renal function between the contralateral and operative sides was > 10% in 79 patients (84.9%). Among those in the group with abnormal renal function, 42 (53.2%), 31 (39.2%), and 6 (7.6%) patients showed stability, aggravation, and improvement in renal function at postoperative 36 months, respectively. The positive relationship between surgery and postoperative separate renal function in this study is consistent with previous studies [16–18]. However, other studies measuring renal function 1 year postoperatively reported stability in 70.2% of cases and aggravation in 17.5%, showing a lower proportion of stability and a higher proportion of aggravation compared to our findings [10]. The long-term 3-year follow-up data shows that 42.1% of patients experienced renal function aggravation, compared to the 1-year follow-up data. A key point of this study is that it is important to explain to patients who do not show recovery within 1 to 2 years that there is a 42.1% chance their renal function may deteriorate over time.

In this study, no significant difference was found in renal functional outcomes between RIRS and mini-PCNL for both the group that was followed up for all 36 months and the group that showed normal results and was followed up only until the 1 st or 2nd year. The results also showed that mini-PCNL may be safer than RIRS in patients who were followed up for up to 3 years. These findings are consistent with previous research on postoperative renal function outcomes following RIRS and mini-PCNL procedures. Unlike conventional PCNL, mini-PCNL utilizes smaller nephrostomy tracts, potentially reducing kidney damage, which may explain the lack of significant difference in kidney damage from mini-PCNL.

There have also been studies on predictors of postoperative renal function aggravation. Kukreja et al. reported high preoperative serum creatinine level, renal cortical atrophy, larger stone burden, proteinuria greater than 300 mg/d during follow-up, history of nephrolithiasis, presence of hydronephrosis, and recurrent urinary infection as significant predictors of renal function deterioration [17]. However, this study was limited by its reliance on serum creatinine levels to asess renal function based on creatinine levels. Piao et al. evaluated relative renal function using ^99 m^Tc-DTPA or ^99 m^Tc-DMSA at 3 months postoperatively and found that the presence of hydronephrosis and three or more stones were significant predictors of renal function deterioration [9]. Jung et al. assessed renal function using ^99 m^Tc-DTPA at 1 year postoperatively, where high preoperative serum creatinine levels and a history of previous stone procedures showed borderline significance in renal function deterioration [10]. In this study, undergoing mini-PCNL, having higher preoperative creatinine levels, the presence of preoperative hydronephrosis, and S-ReSC > 3 were significant predictors of renal function aggravation. This study evaluated renal function using ^99 m^Tc-DTPA at 3 years postoperatively, making it significant for having the longest follow-up data available to date.

Several studies have examined long-term renal function changes after conventional PCNL. Akman et al. reported that eGFR improved or remained stable in patients undergoing PCNL for staghorn calculi with follow-up beyond one year [18]. Bilen et al. found similar results in CKD patients, suggesting PCNL may be safe even in those with impaired baseline renal function [19]. El-Nahas et al. also reported stable renal function after long-term follow-up based on eGFR assessment [20]. By integrating findings from both minimally invasive procedures (mini-PCNL, RIRS) and conventional PCNL, our study offers a comprehensive perspective on postoperative renal function changes. The extended follow-up period further reinforces the clinical significance of these predictors, emphasizing the importance of individualized patient selection and long-term monitoring.

In this study, a 10% threshold was chosen based on previous studies from our group, which consider a 10% variation as a meaningful change [9, 10]. Given the inherent fluctuations of ^99 m^Tc-DTPA scintigraphy, this threshold helps distinguish true functional changes from measurement errors. While sensitivity analysis could offer further insights, maintaining this cut-off ensures consistency and comparability with prior research.

The limitations of this observational study include a relatively small sample size and potential selection bias, as well as the uneven distribution of patients between the mini-PCNL (32 patients) and RIRS (61 patients) groups, which may affect statistical comparisons. Although this study spanned a 3-year follow-up period, which is currently the longest follow-up data available, longer-term outcomes may provide further insights into the durability of renal functional changes. Additionally, renal function in this study was assessed without incorporating additional biomarkers such as KIM- 1, which could provide a more comprehensive evaluation. Future studies should consider integrating multiple assessment methods to enhance the accuracy of renal function analysis.

The differences in stone volumes between the RIRS and mini-PCNL groups may introduce confounding factors, as they reflect differences in surgical indications. The authors attempted to adjust for these differences through multivariate analysis, accounting for variations in stone volume. Furthermore, urinary infection, metabolic syndrome, and hyperuricemia, which are known to influence long-term renal function, were not explicitly analyzed as independent variables in this study. Additionally, other potential contributors such as hypertension, dietary factors, and recurrent stone formation were not comprehensively assessed. The impact of multiple surgeons performing RIRS, as opposed to a single surgeon performing mini-PCNL, may also introduce variability in outcomes. These factors should be considered in future research to provide a more comprehensive understanding of renal function changes post-surgery.

## Conclusion

This study demonstrated that mini-PCNL and RIRS offer similar renal function outcomes over a 3-year follow-up period. A key point of this study is that it is important to explain to patients who do not show recovery within 1 to 2 years that there is a 42.1% chance their renal function may deteriorate over time. Significant predictors of renal function aggravation include high preoperative creatinine levels, preoperative hydronephrosis, and S-ReSC > 3. The study’s 3-year follow-up is the longest available data, but further research is needed to understand the longer-term effects of these procedures.

## Data Availability

Due to legal and ethical restrictions, our raw data cannot be shared publicly.
